# Comparative analysis of SPL transcription factors from streptophyte algae and embryophytes reveals evolutionary trajectories of *SPL* family in streptophytes

**DOI:** 10.1038/s41598-024-51626-2

**Published:** 2024-01-18

**Authors:** Alisha Alisha, Zofia Szweykowska-Kulinska, Izabela Sierocka

**Affiliations:** Department of Gene Expression, Faculty of Biology, Institute of Molecular Biology and Biotechnology, Adam Mickiewicz University, Uniwersytetu Poznanskiego 6, 61-614 Poznan, Poland

**Keywords:** Evolution, Plant sciences

## Abstract

*SQUAMOSA-PROMOTER BINDING PROTEIN-LIKE* (*SPL*) genes encode plant-specific transcription factors which are important regulators of diverse plant developmental processes. We took advantage of available genome sequences of streptophyte algae representatives to investigate the relationships of *SPL* genes between freshwater green algae and land plants*.* Our analysis showed that streptophyte algae, hornwort and liverwort genomes encode from one to four *SPL* genes which is the smallest set, in comparison to other land plants studied to date. Based on the phylogenetic analysis, four major *SPL* phylogenetic groups were distinguished with Group 3 and 4 being sister to Group 1 and 2. Comparative motif analysis revealed conserved protein motifs within each phylogenetic group and unique bryophyte-specific motifs within Group 1 which suggests lineage-specific protein speciation processes. Moreover, the gene structure analysis also indicated the specificity of each by identifying differences in exon–intron structures between the phylogenetic groups, suggesting their evolutionary divergence. Since current understanding of *SPL* genes mostly arises from seed plants, the presented comparative and phylogenetic analyzes from freshwater green algae and land plants provide new insights on the evolutionary trajectories of the *SPL* gene family in different classes of streptophytes.

## Introduction

*SQUAMOSA PROMOTER BINDING PROTEIN-LIKE* (*SPL*) genes encode plant-specific transcription factors (TFs) that are widely distributed from unicellular green algae to angiosperms^1^. For the first time they were described in *Antirrhinum majus* (snapdragon), based on their ability to specifically bind to the promoter of floral meristem identity gene *SQUAMOSA* (*SQUA*), an orthologue of *APETALA1* gene from *Arabidopsis thaliana*^2^. SPL proteins are diverse in their primary protein structure but share characteristic SBP domain. The SBP domain is composed of highly conserved 76–78 amino acid residues consisting of two zinc-binding motifs, Cys-Cys-Cys-His and Cys-Cys-His-Cys, respectively. The N-terminal subdomain of SBP-box is composed of extended loops running in an antiparallel manner, followed by two short α-helises while the C-terminal subdomain contains three-stranded antiparallel β-sheet^3^. The zinc ions present in the SBP domain are crucial for its proper folding and stability which is required for recognition and binding to specific DNA sequences^3,4^. The SBP domain binds the cis-element TNCGTACAA^5^, with GTAC as its essential core part^4^. Additionally, a bipartite nuclear localization signal (NLS) motif resides at the C-terminal end of the SBP domain which overlaps with the second zinc-binding motif. This NLS is required for the nuclear import of SPL proteins^4,6^.

In the model angiosperm *A. thaliana,* 16 members in the *SPL* family were identified, whereas in moss, *Physcomitrium patens,* 13 members were found^7–9^. With the progress of sequencing techniques, the identification and evolution of the *SPL* gene family has been widely investigated in angiosperms. Genomic sequencing has revealed 19, 28, 31, and 56 *SPL* genes in *Oryza sativa, Populus trichocarpa, Zea mays* and *Triticum aestivum,* respectively, indicating dynamics of the *SPL* genes evolution within angiosperms^10–13^. However, for the other land plant lineages these studies are heavily underrepresented with only one moss, *P. patens*, being used in comparative analyses^14,15^. Phylogenetic studies have shown that increase in the *SPL* gene number during the evolution of land plants was mainly the result of expansion of genes with 2–10 exons encoding shorter proteins. Moreover, only within this group of *SPLs*, the expression of a large number of genes is regulated by the miR156 and/or miR529 family members through mRNA cleavage and/or translational repression^15^. In *Arabidopsis*, miR156 targets ten members of the *SPL* family while in rice 11 members are targets for miR156/529. SPL proteins regulate different biological processes in angiosperms including vegetative-to-reproductive phase transition, plant height, root development, inflorescence architecture, abiotic stress responses and lateral organs development^16–21^. Whereas in moss *P. patens*, only transcripts of three *SPL* genes are recognized by miR156. Deletion of one of them, Pp*SBP3*, accelerates and increases the number of developing leafy buds from the juvenile protonemal phase, showing that in the wild type plant Pp*SBP3* acts as a negative regulator of moss phase-transition from tip-growing protonema to leafy gametophores^22^. Although not directly comparable due to the life cycle differences of mosses and angiosperms, this function is somewhat similar to At*SPL14*, which functions to delay the transition to adult development^23^.

miR156 is one of the few highly evolutionarily conserved miRNAs in plants^24^. However, miR156 is not found in the microtranscriptome of liverwort, *Marchantia polymorpha*. Instead, miR529 is present as an equivalent module which regulates the transcript level of one of four *SPL* genes, Mp*SPL2*^25,26^*.* Similar to the role of miR156-*SPL* module in seed plants, the miR529c-Mp*SPL2* module was found to regulate the reproductive transition in *M. polymorpha.*^25^. Additionally, for *M. polymorpha* a unique mode of regulation was observed for Mp*SPL1* gene, as it is regulated by liverwort specific miRNA, Mpo-MR-13. The Mpo-MR-13–Mp*SPL* module is implicated in the control of meristem dormancy by light-regulated conditions to modulate the architecture of the thallus branching under shade imitating conditions^26^.

Although the *SPL* gene family has been widely studied in many species, research on the classification and evolution of *SPLs* is still missing from the representatives of non-seed lineages of land plants, hornworts and liverworts, and their closest algal relatives, streptophyte algae. In our study, we took advantage of available genome sequences from the representatives of diverse green plant lineages: streptophyte algae, hornworts, liverworts, mosses, ferns and angiosperms to investigate the phylogenetic relationships of SPL proteins between streptophyte algae and land plants. Furthermore, we have analyzed the SBP domain amino acid conservation among the representatives of each green plant lineage used in our study which was followed by additional protein motifs distribution and exon–intron gene structure analysis. Moreover, the availability of expression data from RNA-sequencing experiments for *A. agrestis, M. polymorpha, P. patens* and *A. thaliana*, allowed us to investigate the expression profiles of *SPL*s in these species. Our study provides substantial insights into understanding the origin and evolution of the *SPL* gene family in embryophytes and emphasizes the importance of studying the biological relevance of *SPLs* in representatives of bryophytes and streptophyte algae.

## Materials and methods

### Identification of *SPL* genes from hornworts and bioinformatic analysis

Genomes with available annotation of two hornwort species, *Anthoceros agrestis* (Bonn) and *Anthoceros punctatus* were downloaded from University of Zurich database^27^. The genome sequence information for *Anthoceros angustus* was downloaded from DYRAD as provided by^28^. The protein sequences of *A. thaliana*, *P. patens* and *M. polymorpha* were retrieved from the Arabidopsis information resource database TAIR version 10^29^, Phytozome version 13^30,31^ MarpolBase database, respectively^32^. A total of 16 *A. thaliana*, 13 *P. patens* and four *M. polymorpha* SPL protein sequences were used as queries to identify putative SPL protein sequences from *A. agrestis*, *A. punctatus* and, *A. angustus* by using local BLASTP (Table S1). An e-value of < 10^–5^ and bit-score > 100 was used as an initial cut-off to claim significant matches, remove redundant hits and select unique sequences for further analysis. In order to ensure the presence of SBP domain, all the candidate SPL proteins were searched against SMART^33^ and ScanProsite databases^34^.

The miRNA binding sites were identified in the hornworts *SPL* gene transcripts using psRNATarget server^35^. The molecular weight (Mw) and theoretical isoelectric point (p*I*) of *Anthoceros* SPL protein sequences were calculated using Compute pI/Mw tool in the ExPASy server^36,37^. The subcellular localization was predicted online by WoLFPSORT^38,39^.

### Phylogenetic tree construction

In order to identify phylogenetic relationships between SPL proteins across streptophytes, representatives of freshwater green algae and land plants were selected. In the evolutionary context, extant streptophyte algae can be divided into two grades, the lower-branching KCM-grade, consisting of the Klebsormidiophyceae, Chlorokybophyceae, and Mesostigmatophyceae, and the higher-branching ZCC-grade consisting of the Zygnematophycaneae, Coleochaetophyceae, and Charophyceae^40^. Therefore, for our analysis we included the representatives of both clades, *Chlorokybus atmophyticus* and *Klebsormidium nitens* from the lower branching grade, and, *Chara braunii* and *Zygnema circumcarinatum* from the higher branching grade. From embryophytes, representatives of liverworts (*M. polymorpha, Marchantia paleacea* and, *Metzgeria crassipilis*), mosses (*P. patens, Ceratodon purpureus* and, *Sphagnum fallax*), hornworts (*A. angustus, A. agrestis* and, *A. punctatus*), ferns (*Ceratopteris richardii*) and, angiosperms (*Amborella trichopoda, A. thaliana* and, *O. sativa*) were chosen for phylogenetic tree construction. The SPL protein sequences from *Chlamydomonas reinhardtii, C. purpureus, S. fallax. C. richardii, A. trichopoda* and, *O. sativa* were retrieved from Phytozome version 13^30,41^. The SPL protein sequences from streptophyte algae species were retrieved from Phycocosm^42,43^. The SPL protein sequences of *M. paleacea* and *M. crassipilis* were obtained from NCBI and 1KP databases, respectively^44–47^. The full length SPL protein sequences were aligned using CLUSTALW tool in MEGA11^48^. Further, the phylogenetic tree was constructed by using bootstrap maximum likelihood method with 1000 replicates to obtain support values for each branch. CRR1 protein from chlorophycean algae representative, *C. reinhardtii* was used as an outgroup^49,50^.

### Gene structure analysis and conserved protein motifs characterization

The exon–intron structures of *SPL* genes were analyzed by GSDS software^51^. Conserved motif analysis in SPL proteins was performed using MEME program (Multiple EM for Motif Elicitation’ v5.4.1)^52^. The number of predicted motifs was set to 20 with the default parameters (minimum width 6 and maximum width 50). All putative *C. reinhardtii* SPL sequences were queried against SMART^33^ and ScanProsite databases^34^ to confirm the conserved SBP domain presence. Only eight sequences containing the conserved two zinc-binding sites, Cys-Cys-Cys-His and Cys-Cys-His-Cys, were selected for further analysis. The sequence logo for SBP domain sequences was generated by WebLogo 3 platform^53^.

### Cis-acting element analysis of *SPL* gene promoters

The 1500 bp upstream sequences from the start codon for each *SPL* gene sequences from *M. polymorpha, P. patens* and *A. thaliana* were retrieved from the respective genomic resources. For the two hornwort species (*A. agrestis* and, *A. punctatus*), bedtools were used to retrieve 1500 bp upstream sequences for each *SPL* gene^54^. The putative *cis*-elements were identified using PlantCARE software^55^. The identified motifs shown to be putatively involved in plant growth and development, light responsiveness, stress and phytohormone responses are summarized in this study (Table S4).

### Expression profiling of *SPL* genes

The expression data for *A. thaliana* and *P. patens* were downloaded from expression atlas, EMBL-EBI and PEATmoss database, respectively^56–58^. The expression data for *M. polymorpha* and *A. agrestis* (Bonn) were obtained from studies published by^59^ and^60^, respectively. The detailed description of the RNA-seq datasets used in our analysis is provided in Table S5. A heat map presenting the expression profiles of *SPL* genes for each plant was generated using RStudio^61^.

## Results

### Identification of *SPL* genes from three hornwort genomes

BLASTP was used to identify the *SPL* genes from three hornwort genomes *A. angustus*, *A. agrestis* and *A. punctatus*, while SMART and ScanProsite tools were used to validate the results^62,63^. After removing the redundant sequences and sequences with incomplete SBP-box domain, four *SPL* genes were identified in the genomes of *A. agrestis* and *A. punctatus* which were named Aa*SPL1-4* and Ap*SPL1-4,* respectively (Table 1). The gene nomenclature of the identified hornwort *SPL* genes was carried out on the basis of their identity with the respective four members of *M. polymorpha SPL* family^64^. In the case of *SPL* family from *A. angustus,* three genes, An*SPL2-4,* were identified that encode SPL proteins with complete SBP domain. Moreover, one additional protein was found with 81.33% identity to ApSPL1 and 84.68% identity to AaSPL1 protein sequences, however missing the SBP domain. Therefore, the gene was named as An*SPL1-like* and excluded from our further analysis.

**Table 1:** The characteristics of *SPL* genes identified in three hornwort species. Aa—*Anthoceros agrestis*, An—*Anthoceros angustus*, Ap—*Anthoceros punctatus*.

Gene name^a^	Gene ID^b^	Transcript^c^	miR156/529c target site^d^	CDS^e^ (bp)	Protein^f^ (aa)	Mw^g^ (kDa)	pI^h^	Subcellular localization^i^
Aa*SPL1*	AagrBONN_evm.model.Sc2ySwM_344.856	Aa*SPL1*	No	774	257	27.67	10.2	Nucleus
Aa*SPL2*	AagrBONN_evm.model.Sc2ySwM_344.857	Aa*SPL2*	Yes	1611	536	57.08	9.11	Nucleus
Aa*SPL3*	AagrBONN_evm.model.Sc2ySwM_344.2221	Aa*SPL3*	No	1395	464	49.92	7.33	Nucleus
Aa*SPL4*	AagrBONN_evm.model.Sc2ySwM_369.244	Aa*SPL4*	No	2787	928	101.12	6.02	Nucleus
An*SPL1-like*	AANG003444	An*SPL1-like*	No	888	295	305.8	5.52	Nucleus
An*SPL2*	AANG003445	An*SPL2*	Yes	2652	883	93.19	8.70	Nucleus
An*SPL3*	AANG008387	An*SPL3*	No	2409	802	87.938	8.99	Chloroplast/Nucleus
An*SPL4*	AANG000675	An*SPL4*	No	2955	984	106.27	6.04	Nucleus
Ap*SPL1*	Apun_evm.model.utg000107l.74	Ap*SPL1.1*	No	2367	788	83.62	8.87	Nucleus
		Ap*SPL1.2*	No	2397	798	84.65	8.87	Nucleus
Ap*SPL2*	Apun_evm.model.utg000107l.75	Ap*SPL2.1*	Yes	1746	581	61.2	8.98	Nucleus
		Ap*SPL2.2*	Yes	2616	871	91.71	8.83	Nucleus
Ap*SPL3*	Apun_evm.model.utg000185l.396	Ap*SPL3*	No	1383	460	49.54	7.33	Nucleus
Ap*SPL4*	Apun_evm.model.utg000116l.202	Ap*SPL4*	No	2895	964	103.7	5.86	Nucleus

^**a**^Name referred to *Anthoceros SPLs* in this work.

^**b**^Gene accession number in database.

^**c**^Transcript name referred to *Anthoceros SPL* Gene ID.

^**d**^Presence of the recognition site for miR156 in *SPL* transcript.

^**e**^Length of coding DNA sequence.

^**f**^Length of deduced SPL protein.

^**g**^Molecular weight.

^**h**^Theoretical isoelectric point.

^**i**^Predicted subcellular localization by WoLFPSORT tool.

The number of splice isoforms for each hornwort *SPL* gene were next analyzed. Only in the case of Ap*SPL1* and Ap*SPL2* genes from *A. punctatus*, two transcript isoforms were annotated for each of these genes. In the case of Ap*SPL1* gene, the two transcript isoforms encode nearly identical proteins with only ApSPL1.2 being 10 amino acids longer at the C-terminus. However, the difference between the Ap*SPL2* gene transcripts were more significant as the shorter isoform encodes ApSPL2.1 protein which is 581 amino acids long while the longer isoform encodes ApSPL2.2 protein 871 aa in length (Fig. S1). Both these protein isoforms are identical at the N-terminal part in which the SBP domain resides but differ notably at their C-terminal ends. It will be important in the future to study the major and minor transcript variants among *A. punctatus SPL* genes. To our further analysis, we selected the longer Ap*SPL1* and Ap*SPL2* gene transcript variants (Ap*SPL1.2* and Ap*SPL2.2*) as the encoded proteins showed higher sequence similarity to the MpSPL1 and MpSPL2 proteins than the shorter ones.

The lengths of CDS sequences varied from 774 to 2955 bp while their protein lengths varied from 257 to 984 amino acids (Table 1). The molecular weight of deduced SPL proteins ranged from 27.67 to 106.27 kDa while their isoelectric points ranged from 5.52 to 10.20. The subcellular localization of all hornworts SPL proteins was predicted to be in the nucleus, except AnSPL3 with predicted equal localization values for chloroplast and nucleus. These results have shown the diversity within structural features of *SPL* genes across three hornworts species.

For many plants, it was shown that within the *SPL* family, some of the members undergo post-transcriptional gene expression regulation by conserved miRNAs, miR156 or miR529 and *M. polymorpha* specific Mpo-miR13^65^. While miR156 was identified in the genome of *A. angustus*, no experimental data are available for *A. agrestis* and *A. punctatus* microtransriptomes. Therefore, we applied homology-based search to identify miRNA candidates which could target *A. agrestis* and *A. punctatus SPL* gene transcripts. Mature miRNA sequences from horwnwort, *A. angustus* (miR156) and liverwort, *M. polymorpha* (miR529c and Mpo-miR13) were used as an input sequences^25,26,65,66^. We were unsuccessful in finding sequences matching to miR156/529c or Mpo-miR13 in both *A. agrestis* and *A. punctatus* genomes. That is why we used their *SPL* transcript sequences to predict potential target sites which could be recognized by these miRNAs by using psRNATarget server. Applying a stringent cut-off threshold (maximum expectation from 0 to 2) which reduces the false positive predictions, An*SPL2*, *AaSPL2* and *ApSPL2* mRNAs were recognized as potential targets for miR156 and miR529c (Table S2). However, further experiments are needed to investigate the presence of miRNAs in *A. agrestis* and *A. punctatus* that could regulate Aa*SPL2* and Ap*SPL2* transcripts level. In the case of Mpo-miR13, we did not find any hornwort *SPL* gene which could be under this miRNA regulation.

### Comparative evolutionary analysis of *SPL* gene family across streptophytes

**Figure 1:**
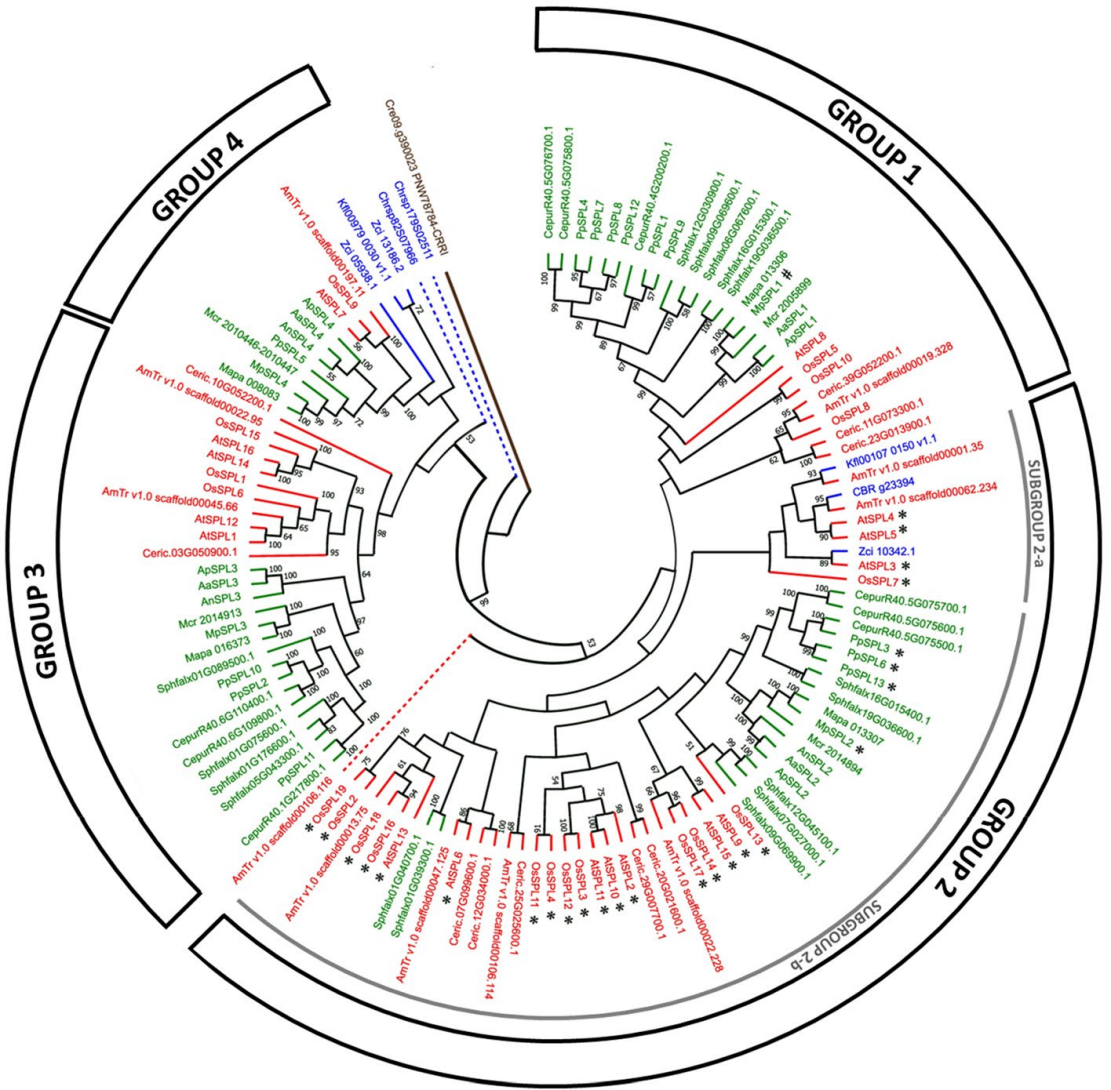
Phylogenetic relationships of SPL proteins from representatives of streptophyte algae (*Chlorokybus atmophyticus, Klebsormidium nitens, Chara braunii* and, *Zygnema circumcarinatum*), liverworts (*Marchantia polymorpha, Marchantia paleacea* and, *Metzgeria crassipilis*), mosses (*Physcomitrium patens, Ceratodon purpureus* and, *Sphagnum fallax*), hornworts (*Anthoceros angustus, Anthoceros agrestis* and, *Anthoceros punctatus*), fern (*Ceratopteris richardii*) and, angiosperms (*Amborella trichopoda, Arabidopsis thaliana* and, *Oryza sativa*)*.* The tree was constructed using the maximum-likelihood method in MEGA 11 software^48^. Number on branches indicates the bootstrap values (%) for 1000 replications; the bootstrap values > 50 are indicated on the nodes. SPL members from the same species are preceded by the prefixes: Chrsp—*Chlorokybus atmophyticus,* Kfl—*K. nitens*, CBR—*C. braunii*, Zci—*Z. circumcarinatum*, Mp—*M. polymorpha,* Mapa—*M. paleacea*, Mcr—*M. crassipilis,* Pp—*P. patens,* Cepur—*C. purpureus,* Sphfalx—*S. fallax,* An—*A. angustus,* Aa—*A. agrestis,* Ap—*A. punctatus,* Ceric—*C. richardii*, AmTr—*A. trichopoda,* At—*A. thaliana* and, Os—*O. sativa*,*.* The CRR1 protein from green algae *Chlamydomonas reinhardtii* was used as an outgroup. *SPL* genes marked by * and ^#^ are regulated by miR156, miR529c and Mpo-miR13, respectively.

To evaluate the evolutionary relationships among SPL proteins in streptophytes, we have built phylogenetic tree based on the multiple sequence alignment of the full length SPL protein sequences from representatives of lower branching streptophyte algae (*C. atmophyticus, K. nitens,*) higher branching streptophyte algae (*C. braunii* and, *Z. circumcarinatum*), liverworts (*M. polymorpha, M. paleacea* and, *M. crassipilis*), mosses (*P. patens, C. purpureus* and, *S. fallax*), hornworts (*A. angustus, A. agrestis* and, *A. punctatus*), ferns (*C. richardii*) and, angiosperms (*A. trichopoda, A. thaliana* and, *O. sativa*). Additionally, CRR1 protein sequence from green algae *C. reinhardtii* was used as an outgroup sequence. The 126 SPL protein sequences from 18 plant species with complete consensus sequence of SBP domain were used to construct the tree (Table 2). From the data presented in Table 2 it is visible that, within the streptophytes, the genomes of streptophyte algae encode the minimal set of SPL proteins, which ranges from one to three, whereas the genomes of embryophytes, hornworts and liverworts, already possess four members of the SPL family. These data may indicate the starting point of evolutionary expansion of the *SPL* gene family in land plants. This expansion might have occurred after the split between mosses and the two remaining clades of bryophytes since more than ten members are already found in the three mosses representatives. Based on the obtained phylogenetic tree, the streptophyte SPL proteins were classified into four distinct groups, Group 1–Group 4, where Group 3 and 4 are classified as sister to Group 1 and 2 with strong support value (Fig. 1). In general, each phylogenetic group contains SPL proteins from all land plant representatives under study with only the exception of Group 4, which lacks a fern representative. Moreover, in all four groups, proteins from bryophytes (Fig. 1 highlighted in green) and tracheophytes (Fig. 1 highlighted in red) grouped as separate subfamilies, respectively, with few exceptions encountered in Group 2. Furthermore, only in Group 2 and Group 4 proteins from streptophyte algae, *K. nitens, Ch. braunii* and *Z. circumcarinatum,* were recognized but not from *Ch. atmophyticus*. Interestingly, the two *Ch. atmophyticus* SPL proteins were not included in any of the identified phylogenetic SPL groups. According to the obtained tree, the Chrsp82S07966 protein is sister to all streptophyte SPL proteins from Group 3 and 4, while Chrsp179S02511 protein is sister to all four groups recognized in our study.

**Table 2:** List of plant species used for constructing phylogenetic tree in Fig. 1.

Species name	Number of SPL proteins
Streptophyte algae	
*Chlorokybus atmophyticus*	2
*Klebsormidium nitens*	2
*Chara braunii*	1
*Zygnema circumcarinatum*	3
Liverworts	
*Marchantia polymorpha*	4
*Marchantia paleacea*	4
*Metzgeria crassipilis*	4
Mosses	
*Physcomitrium patens*	13
*Ceratodon purpureus*	9
*Sphagnum fallax*	17
Hornworts	
*Anthoceros angustus*	3
*Anthoceros agrestis*	4
*Anthoceros punctatus*	4
Fern	
*Ceratopteris richardii*	10
Angiosperms	
*Amborella trichopoda*	11
*Arabidopsis thaliana*	16
*Oryza sativa*	19

In the SPL Group 4, only single gene members are present in the species under study, with the exception of freshwater algae *Z. circumcarinatum* which possess two members in this clade. Therefore, Group 4 has the fewest SPL proteins compared to Group 1–Group 3, which makes it relatively different from the other three groups. Moreover, a distinctive feature of the land plant proteins belonging to this group is the presence of a different signature C4 motif at the first zinc finger structure (Zn-1) in the SBP domain as compared to the canonical C3H motif found in all other SPL proteins. Small and stable number of genes in Group 4 indicates their highly conserved character and resistance to expansion during SPL family evolution (Fig. 1). Interestingly, in addition to lack of fern *C. richardii* SPL Group 4 member, proteins from mosses, *C. purpureus* and *S. fallax,* are also missing. In contrary, the SPL Group 3 is already represented by two *C. richardii* SPLs, while *C. purpureus* and *S. fallax* possess three and four members, respectively. The expansion of the SPL Group 3 protein number is also observed in all angiosperms studied. Only in hornworts and liverworts, single gene members are recognized in Group 3.

The most SPL proteins were observed in Group 2, however with explicit variability in the protein number between different classes of streptophytes. Among representatives of streptophyte algae, hornworts and liverworts, single members of SPL Group 2 were recognized while in mosses already from three to seven proteins belong to this group. Also, in tracheophytes the expansion of SPL Group 2 was observed with five SPLs present in fern *C. richardii* and angiosperm *A. trichopoda* representing sister lineage relative to all other flowering plants, and nine and eleven present in core angiosperm representatives. Interestingly, based on the phylogenetic analysis, Group 2 can be further subdivided into two subgroups, 2-a and 2-b. Subgroup 2-b comprises most of Group 2 SPLs, while Subgroup 2-a contains only nine members. Intriguingly, Subgroup 2-a is composed only from proteins of charophycean algae and angiosperms which might be a consequence of convergent evolution. It is noteworthy, that all *SPL* gene family members from bryophytes and angiosperms described up to date, which are targeted by the conserved miR156 or miR529 are classified within Group 2.

Similar to Group 3, Group 1 contains SPL proteins only from land plants. Group 1 single gene members were recognized in hornworts, liverworts and two angiosperms, *A. trichopoda* and *A. thaliana.* In the genomes of remaining embryophytes, three to six SPL proteins were classified to Group 1. Group 1 *SPL* genes are not under control of miRNA, except the Mp*SPL1* gene from the liverwort *M. polymorpha* which is targeted by Marchantia-specific Mpo-mr-13^26,64^*.*

**Figure 2:**
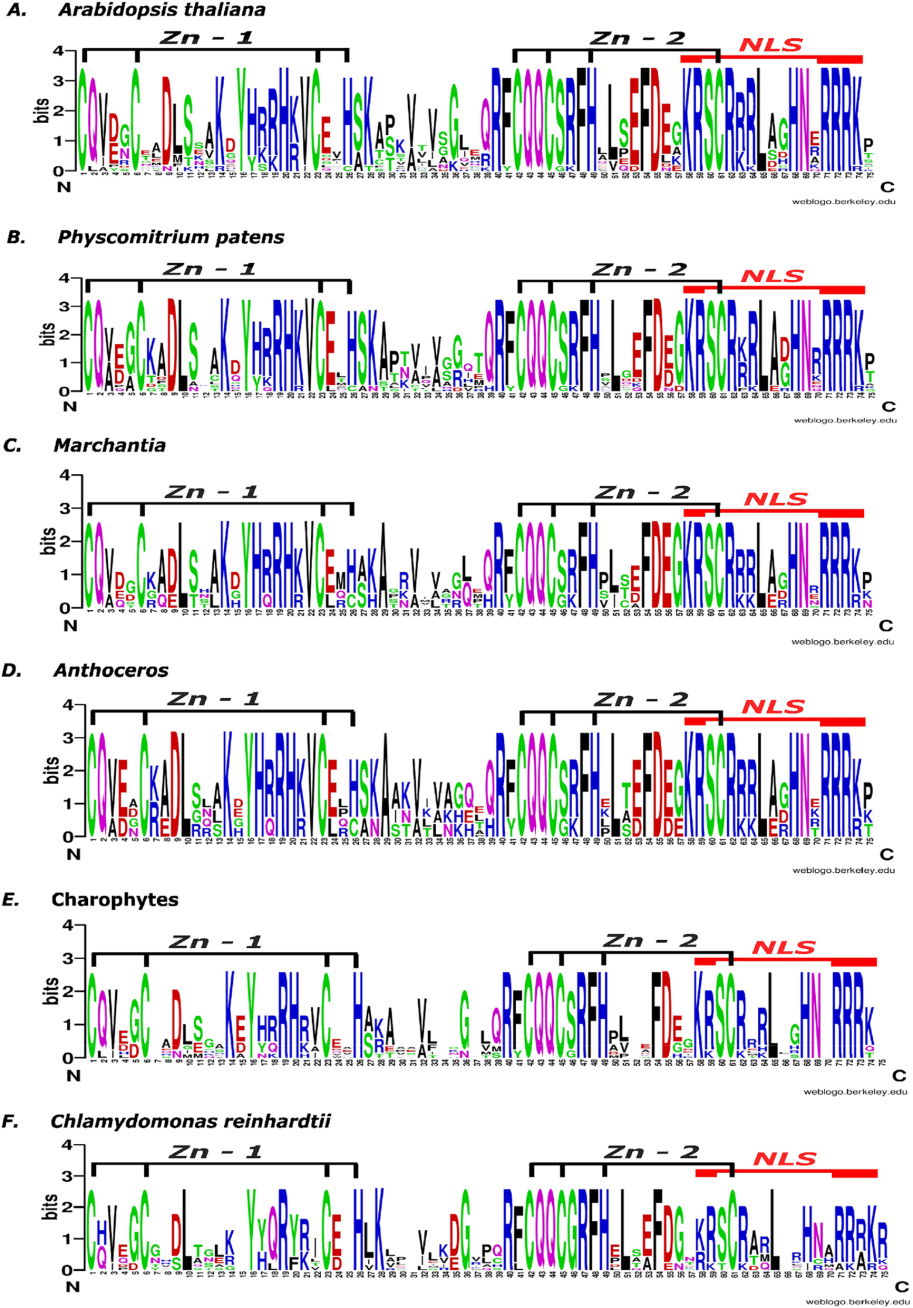
Sequence logo of conserved SBP domain of SPL proteins from (**a**) angiosperm *A. thaliana*, (**b**) moss *P. patens*, (**c**) two liverworts, *M. polymorpha and M. paleacea*) (**d**) three hornworts, *A. agrestis*, *A. punctatus* and *A. angustus*), (**e**) four streptophyte algae, *C. atmophyticus*, *K. nitens*, *C. braunii* and, *Z. circumcarinatum*, and (**f**) chlorophyte *C. reinhardtii*. The weblogo includes 16 SBP sequences from *A. thaliana*, 13 from *P. patens*, four each from *M. polymorpha* and *M. paleacea*, three from *A. angustus*, four each from *A. agrestis* and A. punctatus, two from *C. atmophyticus,* two from *K. nitens,* one from *C. braunii*, three from *Z. circumcarinatum*, and ten from *C. reinhardtii*, respectively. Zn-1—zinc finger structure 1, Zn-2—zinc finger structure 2, NLS—nuclear localization signal. The sequence logo was generated using Weblogo online software^53^. The overall height of the stack reflects the extent of sequence conservation at that position, and the height of the letters within each stack indicates the relative frequency of each amino acid at that position.

As only the SBP domain was found to be conserved and shared between SPL proteins across the green plants lineage, we further analyzed the conservation of each amino acid residue for chlorophytes, streptophyte algae, hornworts, liverworts, mosses and angiosperms representative by using Weblogo tool (Fig. 2). All of the SBP domains from analyzed species shared conserved zinc-binding amino acid residues in the two zinc finger-like structures, Zn-1 and Zn-2, and the bipartite nuclear localization signal (NLS). In the case of chlorophytes and streptophyte algae representatives, the amino acids across the Zn-2 site showed similar conservation when compared to land plants (Fig. 2E,F). However, the amino acids in the Zn-1 region are significantly less conserved with characteristic positions that differ from those observed in land plants. The *C. reinhardtii* first zinc finger region lacks the well conserved basic amino acid residues present in land plants at positions 17–21 from which only arginine (at position 19) is present in this green algae. While for streptophyte algae, the sequence conservation from positions 17–21 is more prevalent than *C. reinhardtii* but lower than land plants. Similarly, higher divergence was observed in the nuclear localization signal (positions 71–74) at the C-terminal end of the SBP domain in *C. reinhardtii* than in streptophyte algae, when compared with land plants. In the case of hornworts and liverworts, the SBP domain from these bryophytes resembles more that of *A. thaliana* than streptophyte algae and chlorophytes. Moreover, this analysis showed that the conservation of amino acids at the functional sites of the SBP domain increased during the evolution of land plant SPL proteins. Taken together, the phylogenetic results and the SBP domain conservation analysis suggest that *SPL* genes predate the origin of land plants and the SBP domain from algae and land plants originated from a common ancestor.

### Identification of conserved motifs in SPL proteins

**Figure 3:**
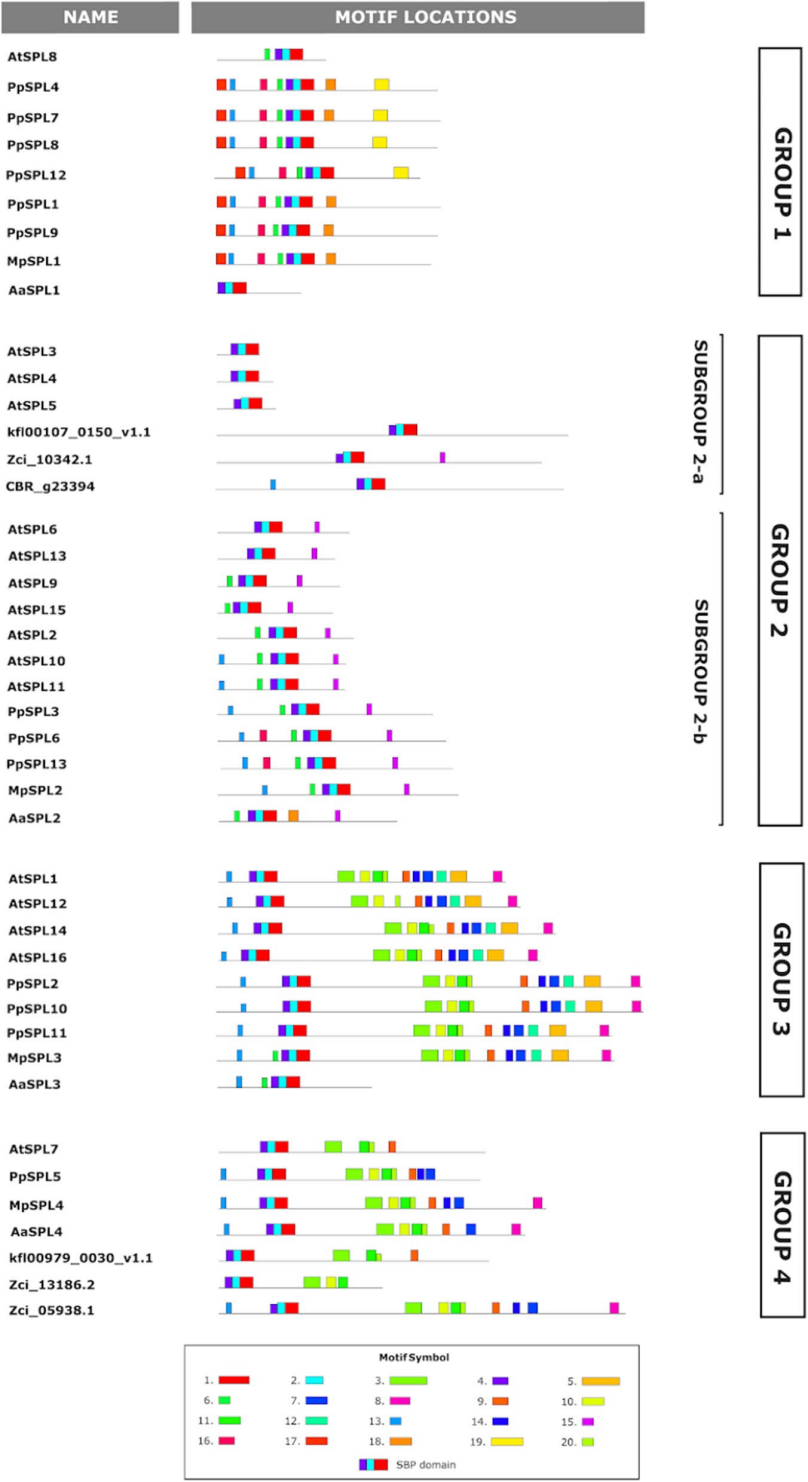
Conserved motifs in SPL proteins from *K. nitens*, *C. braunii* and, *Z. circumcarinatum* (streptophyte algae representatives), *A. agrestis* (hornworts representative), *M. polymorpha* (liverworts representative), *P. patens* (moss representative), and,*A. thaliana* (angiosperms representative). The motif search was performed using MEME online tool^52^ with full length protein sequences as a query. SPL proteins are grouped according to their phylogenetic relationships. Different motifs are represented with colors shown in the legend. Motifs 1, 2 and 4 with red, blue and violet color denote SBP-box domain which is conserved amongst all SPL proteins. The consensus sequence of each motif is presented in Table S3.

To analyze the diversity and similarity between SPL protein structures from streptophyte algae, bryophytes and angiosperms, conserved domains and motifs were identified using MEME online tool^52^. During this analysis, we have focused on SPL proteins which were classified to each phylogenetic group from all studied freshwater algae and single representatives of hornworts (*A. agrestis*), liverworts (*M. polymorpha*), mosses (*P. patens*) and angiosperms (*A. thaliana*). The co-ordinates and sequences of SBP-box domains within each SPL protein were obtained using Pfam 35.0 database^67^. A conserved SBP domain was found in all SPL members, represented by Motifs 4, 2, and 1 after MEME analysis (Fig. 3). Additionally, several conserved motifs were also present in the proteins belonging to the same phylogenetic group (Fig. 3). For example, Motifs 16–19 seem to be bryophyte-unique motifs found only in members of Group 1 proteins (with the exception of motif 18 present in *A. agrestis* Subgroup 2-b protein, and motif 16 present in two *P. patens* proteins from Subgroup 2-b), indicating that these motifs might be important for controlling some lineage specific processes (Figs. 3, S2). Based on the protein length, Group 1 can be further divided in two subgroups: (i) longer proteins represented by all bryophytes Group 1 SPL proteins (with the exception of hornwort AaSPL1 protein) and fern *C. richardii* Group 1 SPL proteins and (ii) shorter proteins with all Group 1 SPL proteins from angiosperms and *A. agrestis* AaSPL1. Although similar in length to bryophyte proteins, *C. richardii* Group 1 SPL proteins do not exhibit the characteristic arrangement of additional motifs, Motifs 16–19. The origin from a common ancestor and the presence of similar motifs between Group 1 SPL proteins from different classes of bryophytes might indicate the similarity in their biological functions. However, functional studies are needed to test this hypothesis.

In the case of Group 2, relatively low number of protein motifs were found. The majority of Group 2 SPL members possess characteristic motif, Motif 15, composed of 15 aa consensus sequence. Interestingly, the middle part of this protein motif, ALSLLS peptide, represents highly conserved amino acids coded by miR156/529 target site^68^. It might be assumed that all proteins in which Motif 15 was recognized are potential targets for miR156/529 regulation. However, careful inspection of Motif 15 in each identified protein revealed that in the streptophyte alga *Z. circumcarinatum* Zci_10342.1 protein, this consensus peptide is partially conserved, with only four amino acid residues preserved (ALaLLn). Similar situation but concerning single amino acid residue substitution (second leucine in the LL dipeptide is substituted by glycine) was found in two *C. richardii* proteins, Ceric.12G034000.1 and Ceric.07G099600.1. According to known rules for effective miRNA targeting in plants, high miRNA-mRNA complementarity is a requirement for effective gene silencing^69,70^. Any changes in a total number of paired positions between miRNA-mRNA may abolish effective target recognition and lead to suppression of miRNA function. Therefore, the putative miR156/529 regulation sites form *Z. circumcarinatum* and *C. richardii* need experimental verification.

The highest number of motifs were found among Group 3 members. With an exception of two hornwort proteins, AaSPL4 and ApSPL4, all members belonging to Group 3 contain from nine to ten conserved motifs (Motifs 3, 10, 11, 20, 9, 14, 7, 12, 5, 8) (Figs. 3, S2). Additionally, Motif 12 and 5 are specific only for Group 3 SPLs across all land plants used in the study. Interestingly, Motif 5 is composed of ankyrin repeats. The ANK domain has been shown to be associated with protein–protein interactions^71^. What is more, five motifs present in the Group 3 SPLs, namely Motifs 3, 10, 11, 20 and 9, are also present in most SPL proteins from Group 4, both in land plants and streptophyte algae. The high number of similar motifs shared between SPL proteins from different plant species may indicate that these proteins can play similar roles in different plant species or they may possess similar biochemical properties. Taken together, analysis of protein motifs found that SPL proteins from the same phylogenetic groups tend to have similar combinations of protein motifs implying that each phylogenetic group may exhibit the functional conservation, but also underlying the diversity of mechanisms that influenced the SPL family evolution.

### Gene structure analysis of *SPL* genes between streptophyte algae and embryophytes

**Figure 4:**
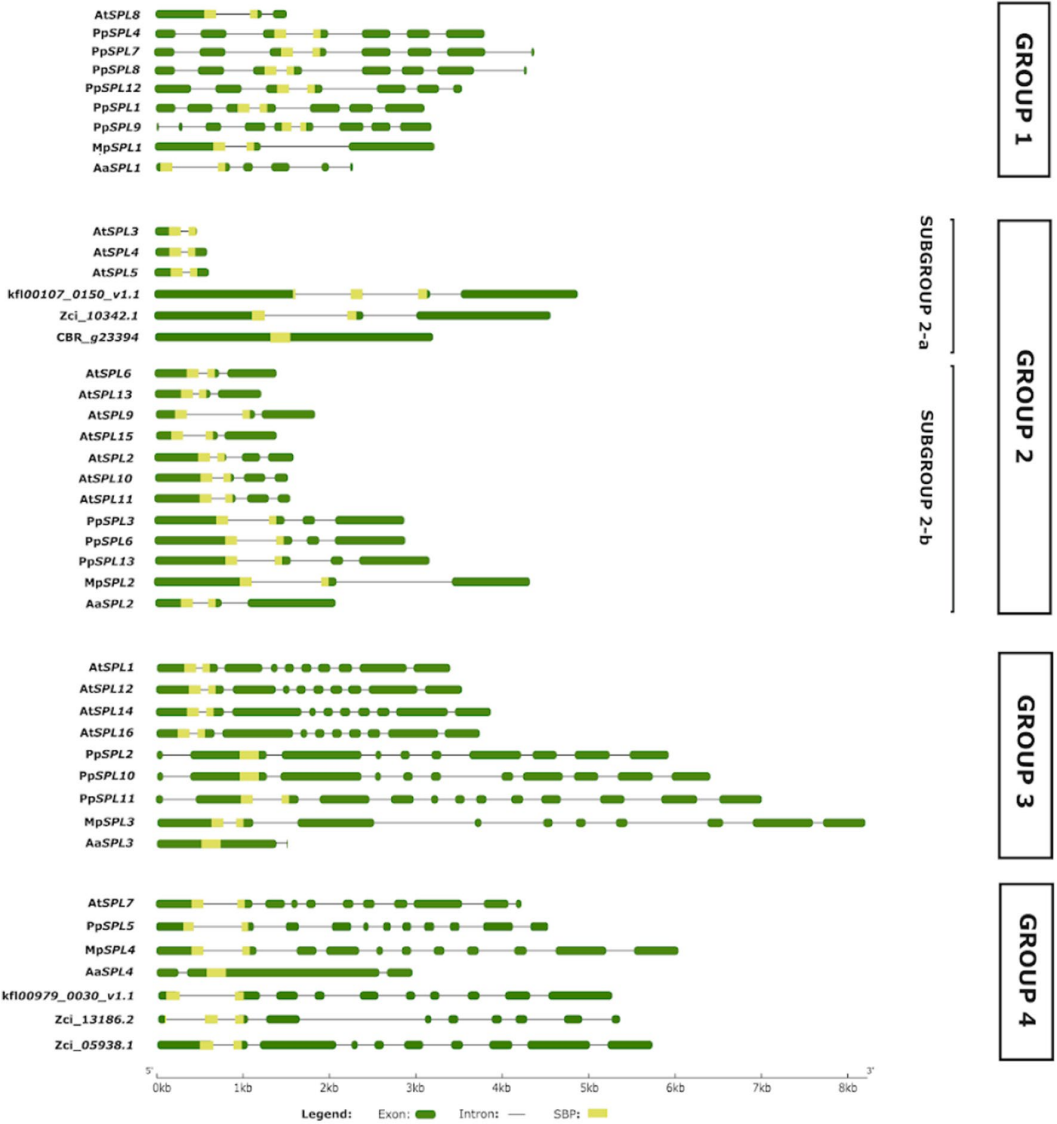
Diagram of exon–intron organization of the *SPL* gene family from *K. nitens*, *C. braunii* and, *Z. circumcarinatum* (streptophyte algae representatives), *A. agrestis* (hornworts representative), *M. polymorpha* (liverworts representative), *P. patens* (moss representative), and, *A. thaliana* (angiosperms representative). The gene structures were analyzed using gene structure display server 2.0^51^ and grouped based on their phylogenetic relationships. In each gene model, exons are shown as green boxes, introns as black lines and SBP-box as yellow rectangular shading. The scale shown at the bottom represents gene lengths in kilobase pairs.

To learn about the structural diversity of *SPL* genes in streptophytes, we performed comparative exon–intron structure analysis of streptophyte algae *SPL* genes with representatives of hornworts (*A. agrestis*) liverworts (*M. polymorpha*), mosses (*P. patens*), and angiosperms (*A. thaliana*). Variations in the number and length of exons and introns were observed in each *SPL* clade (Fig. 4). The highest diversity in the gene exon–intron structure was observed in Group 1, as *M. polymorpha* and *A. thaliana* genes contain two introns, *Anthoceros* four to five introns and *P. patens* six to seven introns. On the other hand, the genes present in Group 2 showed the highest similarity between their gene structures with most genes containing two to three introns. Only one gene from *Ch. braunii* and three genes from *A. thaliana* turned out to be intronless and single-intron genes, respectively (Fig. 4, S3). The members belonging to Group 3 and Group 4, with the exception of *A. agrestis SPLs*, showed the highest number of introns, from eight to ten. The hornworts genes, however, possess only one or two introns in these phylogenetic groups.

Previous studies have shown that the SBP domain of land plants from mosses to angiosperms was encoded by two exons interrupted by an intron with highly conserved position. The splicing site for this intron is located before the dipeptide Phe-His of the conserved CQQC[S/G][R/K]FH octapeptide^72^. Our analysis revealed that this conservation is also true for all *SPLs* from *M. polymorpha,* Group 1 and 2 *SPL* genes from hornworts and most streptophyte algae. Furthermore, the same conservation of intron position was found in the streptophyte alga *Ch. atmophyticus SPL* gene, *Chrsp179S02511,* which according to phylogenetic analysis is sister to all other *SPL* genes from streptophytes used in our study. Only the *SPL* gene from algae *Ch. braunii, SPL* members of Group 3 and 4 from hornworts and two *SPL* genes from moss *P. patens* encode the SBP domain by a single exon. Interestingly, two streptophyte algae genes, *Kfl00107_0150_v1.1* and *Zci_13186.2*, from *K. nitens* and *Z. circumcarinatum,* respectively*,* possess additional intron at the very beginning of SBP-coding region. In both cases the splicing site is located before the tetrapeptide V[E/D]GC. This feature may indicate that in streptophyte algae genomes some members of *SPL* family underwent random insertions of introns within the SBP-coding region.

Based on the identified exon–intron structures of *SPL* genes, differences in the intron lengths were observed, especially for the hornworts *SPL* genes. To validate these differences, we calculated the average intron lengths of the *SPL* genes for each bryophyte species and *A. thaliana*. The obtained values for *A. thaliana*, *P. patens*, *M. polymorpha* and *A. agrestis SPL* genes were 51 bp, 156 bp, 275 bp and 104 bp, respectively showing that *A. thaliana* and hornworts *SPL* genes possess the shortest introns, while *M. polymorpha* exhibits the longest introns from all the analyzed *SPL* genes. These data coincide with the data published for the genome of each plant studied, where the average intron lengths were calculated to be 164 bp in *A. thaliana*, 278 bp in moss *P. patens*, 392 bp in liverwort *M. polymorpha* and 104/103 in hornworts^32,60,73,74^. The specificity of the intron length and number within *SPL* genes in both *A. agrestis* species correlates with the high gene density in their genomes, which is achieved by the presence of many intron-less genes. Additionally, the gene structure of these *SPL* genes reflects a characteristic feature of both hornwort genomes which is the presence of three to four exons per gene on average^60,75^.

### Analysis of *cis*-elements in promoter regions of *SPL* genes

**Figure 5:**
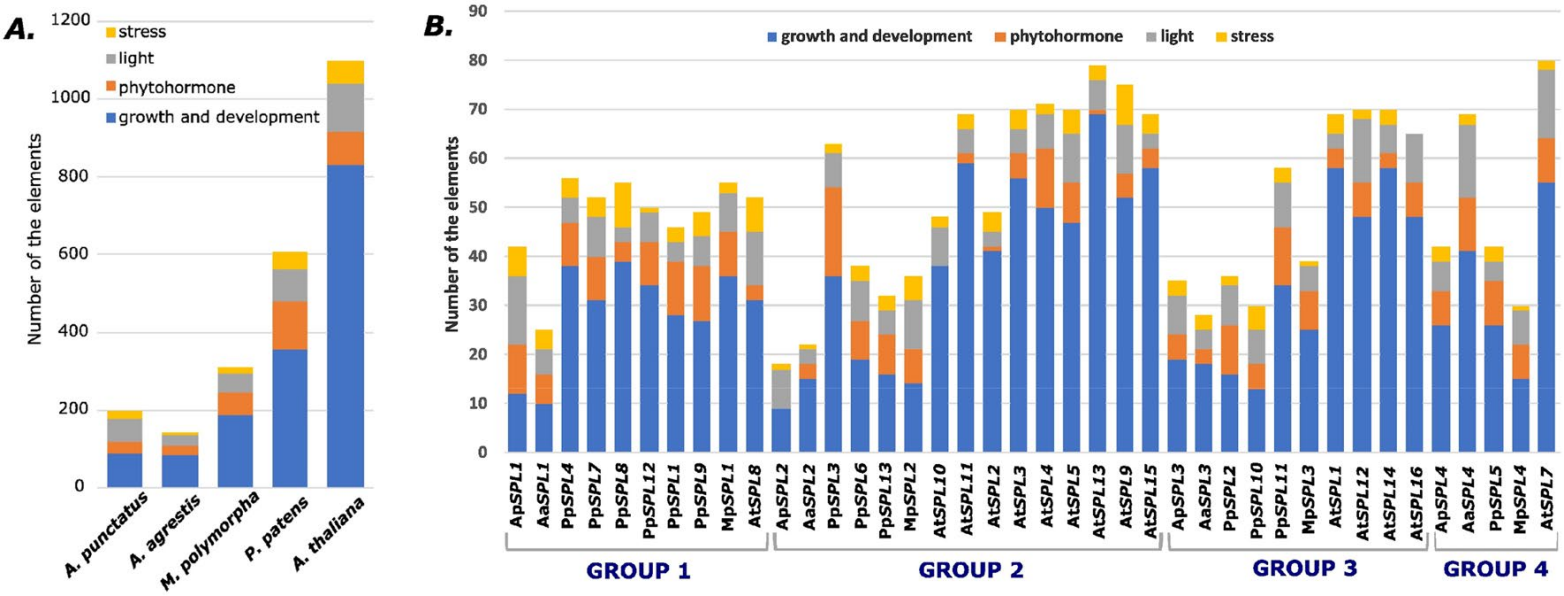
*Cis*-elements analysis of the investigated *SPL* genes from representatives of bryophytes and dicots. (**A**) The number of *cis*-elements in the promoter regions of *A. agrestis, A. punctatus, M. polymorpha, P. patens,* and *A. thaliana*, *SPL* genes. (**B**) The number of *cis*-elements in each *A. agrestis, A. punctatus, M. polymorpha, P. patens,* and *A. thaliana SPL* gene promoter region grouped according to their phylogenetic relationships. The regulatory elements were detected in the 1500 bp sequences upstream of the start codon of each *SPL* gene using PlantCARE database^55^. The elements associated with specific functions are denoted by different colors for each gene. The detailed information concerning the *cis*-elements analysis is given in Table S4.

*Cis*-elements in the promoter region play important roles in the gene transcription regulation and as an adaptive mechanism to respond to different environmental conditions^76^. To study the potential transcription regulation signals, *cis-*regulatory elements were identified in the promoter regions of investigated *SPL* genes using PlantCARE database (Table S4). A large number of *cis-*elements were detected and further classified into four subdivisions: growth and development, phytohormone response, light responsiveness and stress response (Fig. 5A,B, Table S4).

More than half of predicted *cis*-elements, including A-box, CAAT-box, CAT-box, CCAAT-box, GCN4 motif, NON-box, O2 site, RY element, TATA-box, AT-rich elements and circadian clock-related elements were classified under growth and development category in all studied plant species. The number of growth and development elements increased with increase in diversity of plant species. Several phytohormone responsive elements, including ABRE, AuxRR-core, CGTCA-motif, GARE-motif, TGA element, P-box, HD-Zip 3, TATC-box, TCA-element, TGA-box and TGACG-motif were identified in all four lineages. The highest number of phytohormone response elements were identified in moss and the lowest in hornworts. In the light responsive category, many elements were identified with mainly Sp1, G-box, TCT-motif and TCCC-motif being enriched. The highest number of light responsive elements were identified in *A. thaliana*. Furthermore, the identified stress response elements included ARE, TC-rich repeats, GC-motif, LTR and MBS were most common and highest in moss and dicot. In two examples, Ap*SPL2* from *A. punctatus* and At*SPL10* from *A. thaliana*, the phytohormone responsive elements were not detected (Fig. 5B). Also, the absence of stress response elements in the promoter region of At*SPL16* was observed. These results showed that *SPL* genes from different phylogenetic groups and plant species possibly participate in diverse physiological processes, developmental regulation, and abiotic stress responses.

### *SPL* expression profiles across different tissues in *Arabidopsis* and bryophytes

**Figure 6:**
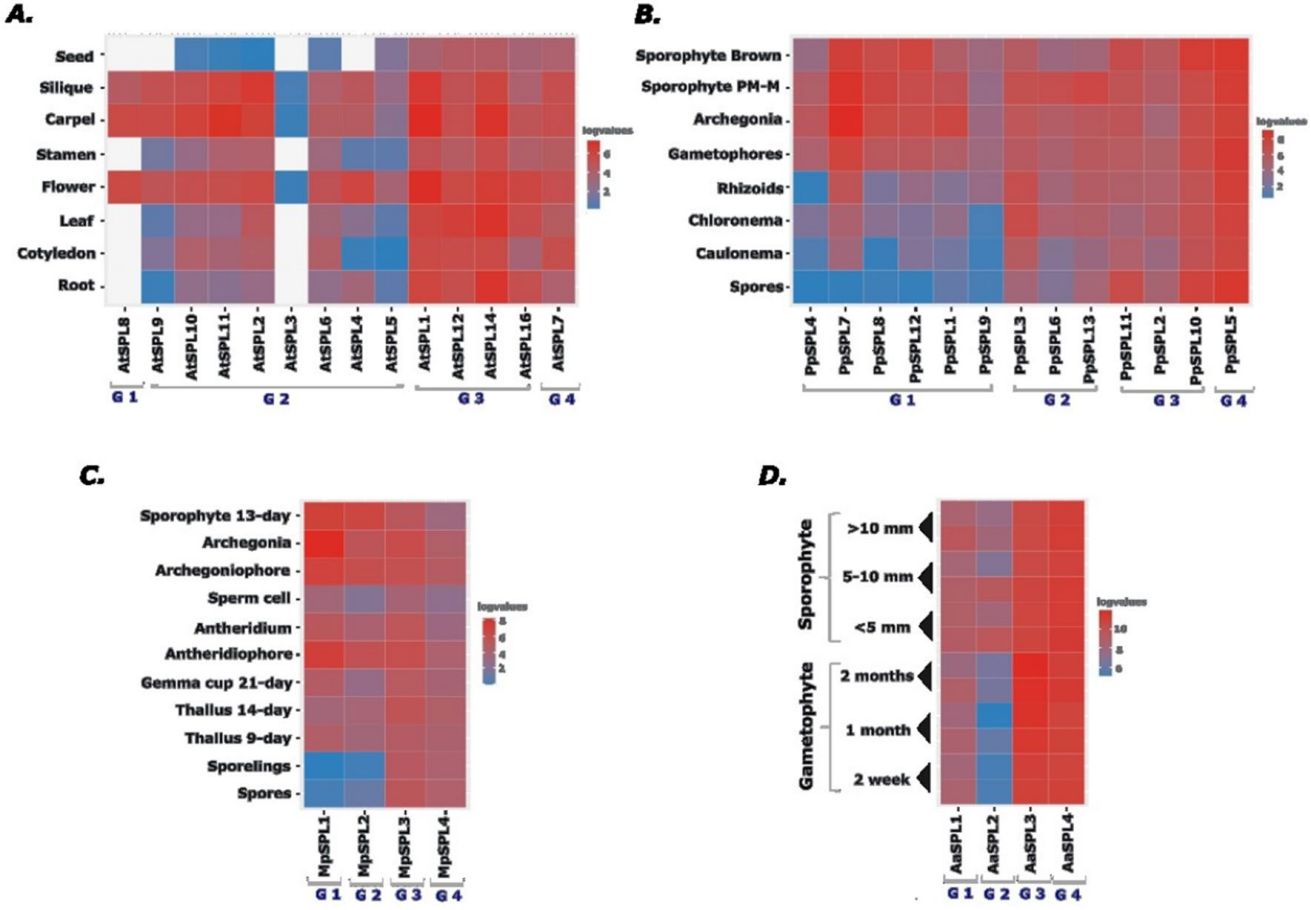
The expression profiles of *SPL* genes from different developmental stages and organs of *A. agrestis, M. polymorpha, P. patens,* and *A. thaliana*. TPM and FPKM values were identified from RNA-seq data and normalized by log2 transformation for: (**A**) *A. thaliana*^77^, (**B**) *P. patens*^58^, (**C**) *M. polymorpha*^59^ and (**D**) *A. agrestis *^60^. The heatmap was generated in RStudio^61^. G1-G4 denotes the names of *SPL* phylogenetic groups, Group 1–4. The red, blue and white colours denote high, low and no expression values.

To have a general overview about the tissue-specific expression profile of *SPL* genes in *A. thaliana* and bryophytes representatives, we gathered the publicly available RNA-seq data for the investigated plant species from different developmental stages and organs to dissect the information about the transcript levels for each *SPL* gene (Table S5). In the case of hornworts, for *A. punctatus* no expression data concerning developmental stages was found and only RNA-seq data for different gametophyte and sporophyte developmental stages of *A. agrestis* was available and used in our analysis^60^. The detected expression levels were plotted as heat maps for each plant species (Fig. 6).

In the case of *A. thaliana,* 14 out of 16 *SPL* genes were expressed in the selected developmental stages (Fig. 6A). Two members belonging to Group 2, At*SPL13* and At*SPL15,* were not detected. According to experimental data showing the expression of AtSPL13 and AtSPL15 fusion proteins tagged with β-glucuronidase in transgenic plants, both these proteins accumulate at very low levels for a short time during leaf development and early stages of inflorescence development, respectively^78^. Most probably such specific expression profiles observed for AtSPL13 and AtSPL15 proteins might be the cause that both these genes are missing in the presented analysis. In *A. thaliana,* the expression patterns of different genes in the same phylogenetic group were observed to be rather similar, suggesting the involvement of *SPL* paralogs in the regulation of similar processes. The most specific expression pattern was observed for Group 1 and correlated mostly with flower development. The *A. thaliana* Group 2 *SPL* genes, although expressed in more developmental stages in comparison to Group 1 *SPL* genes, also exhibited in general enriched expression during flower organs development (Fig. 6A). In turn, the At*SPLs* expression levels from Group 3 and Group 4 were high and at rather similar levels in the analyzed organs and developmental stages. In general, based on their expression pattern, *A. thaliana SPL* genes can be divided into two groups: (i) those with rather constitutive and stable expression levels during all *A. thaliana* developmental stages, and (ii) those showing high expression levels during specific growth and reproduction processes of *A. thaliana* development. Similar division can be observed in moss *P. patens* where the expression data clearly show that Pp*SPL* genes from Group 1 are not expressed or very weakly expressed in spores and protonema while in gametophores and sporophyte their expression level is prominent and stable (Fig. 6B). The *PpSPL7* gene showed the highest expression in archegonia and different stages of sporophyte development what may suggest its importance during moss sexual reproduction and sporophyte maturation. The Pp*SPL* genes from Group 2 showed higher expression during premeiotic to meiotic stages of sporophyte development (sporophyte PM-M) with the exception of Pp*SPL3* which additionally showed high expression in rhizoids and chloronema. The other two *P. patens SPL* groups exhibited constitutive expression in all analyzed moss tissues and developmental stages.

As observed in *A. thaliana* and *P. patens,* also *M. polymorpha* and *A. agrestis SPL* genes belonging to Group 3 and Group 4 exhibited rather constitutive expression profiles in all types of organs and developmental stages analyzed (Fig. 6C,D). In *Marchantia*, *SPL* members belonging to Group 1 and Group 2 showed rather tissue specific expression with the highest expression observed in reproductive organs development and in young sporophyte. This finding may indicate that the Mp*SPL* genes are involved in the entire process of growth and development in this liverwort with some additional role for Mp*SPL1* and Mp*SPL2* during sexual reproduction as their expression is up-regulated in *M. polymorpha* sex organs (Fig. 6C). In the case of hornwort *A. agrestis,* the most specific expression pattern was observed for Aa*SPL2* belonging to Group 2 whose expression is mostly found in the sporophyte generation while Aa*SPL1* belonging to Group 1 showed equal expression levels during both gametophyte and sporophyte development (Fig. 6D).

The expression data analysis showed that in all analyzed plant species, the *SPL* genes may fall into one of the two categories in the context of expression profile. First one, genes which are highly expressed in nearly all tissues and that is why may function similarly as housekeeping genes for the maintenance of basal cellular functions (genes from Group 3 and Group 4). What is more, the genes belonging to this category are not regulated by miRNAs. The second category consists of genes with developmentally specified or enriched expression which are important for the regulation of specific processes during growth and reproduction. Importantly, many genes from this category are under post-transcriptional control guided by miRNA (Fig. 1). In three out of four analyzed plant species, including dicot *A. thaliana,* moss *P. patens* and liverwort *M. polymorpha*, *SPL* genes whose expression profile is strongly correlated with sexual reproduction (genes from Group 1 and group 2) were found. Since there is no data concerning gene expression in the reproductive organs of hornwort *A. agrestis,* based on the observed evolutionary conserved mode of action for some representatives within the *SPL* family, it might be hypothesized that most probably also in *A. agrestis* at least one of the *SPL* family members could be engaged in the regulation of the reproductive pathway.

## Discussion

*SPL* genes form a major family of plant-specific transcription factors and encode proteins with a highly conserved SBP-box DNA-binding domain. They are crucial players regulating different biological processes in plants, including juvenile to adult phase transition, vegetative to reproductive phase transition, apical dominance, flower development and many more^78,79^. In our study, we provide a comparative evolutionary analysis of *SPL* gene family from representatives of different lineages across the plant kingdom, shedding light on their diversity, evolutionary relationships, structural features, regulatory mechanisms, and expression patterns.

No SBP-box related sequences for hornworts were available in the public databases at the time we started our attempt to identify SBP-box genes from this plant lineage. Firstly, our investigation involved identifying *SPL* genes in the three hornwort genomes: *A. angustus*, *A. agrestis* and *A. punctatus*. In our study, four *SPL* genes were identified in two hornwort species, *A. agrestis* and *A. punctatus* what is similar to the set of *SPL* genes observed in the liverwort *M. polymorpha*^64,60^. In another hornwort, *A. angustus*, three *SPL* genes were identified and one *SPL-like,* because of the absence of SBP domain (Table 1). The missing N-terminal with SBP domain in AnSPL1-like might be because of the first annotation of currently available *A. angustus* genome. The identification of *SPL* genes in the three hornwort genomes showed diversity in transcript isoforms and structural features highlighting the complexity of *SPL* gene family within three hornworts species.

The evolutionary analysis across streptophytes classified the SPL family into four major groups: Group 1- Group 4 (Fig. 1). Notably, Group 1 and 2 appear as sister groups to Group 3 and 4 what is supported by high confidence level. Interestingly, within streptophytes, streptophyte algae, liverworts and hornworts encode a minimal set of SPL proteins, showcasing a restricted *SPL* gene repertoire in the freshwater algae and early branching land plants. The identification of only one to four *SPL* members in streptophyte algae, hornworts and liverworts representatives as compared to other land plants underlines that the evolution of all land plant *SPL* genes was a result of several rounds of gene duplication and next speciation events of the paralog genes. Overall, this comparative phylogenetic analysis provides us with an understanding of evolutionary trajectories and diversification of SPL family across the streptophytes.

Moreover, very high amino acid conservation was found within the SBP domain of land plants, in particular for the zinc-finger like structures and the NLS signal (Fig. 2A–D). As shown in structural studies using *A. thaliana* SPL proteins, all conserved basic amino acids from Zn-1, Zn-2 and NLS signal form a positively charged surface involved in binding the negatively charged DNA^3^. Although SPL proteins were also described in algal representatives, their SBP domains showed lower degree of conservation in the amount of basic amino acids, especially within the first zinc-finger like structure (Fig. 2E,F). In fact, Birkenbihl and co-workers have shown that *C. reinhardtii* CRR1 protein exhibited a significantly lower affinity to the *A. thaliana*-derived 15 bp *AP1* promoter fragment and to the *C. reinhardtii*-derived copper response element (CuRE) in comparison to *A. thaliana* AtSPL1, AtSPL3, AtSPL8 and moss PpSPL1 proteins^4^. Therefore, the lower amount of basic amino acid in the green algae SBP domain of the CRR1 protein when compared to land plants might be responsible for its lower efficiency to interact with DNA. Among the conserved Arg/Lys residues, those in the N-terminal part of the SBP domain (Lys14, Arg/Lys18, Arg19, Lys/Arg21) are suggested to be the candidate residues that determine the sequence specificity by direct recognition of the DNA bases^3^. All these conserved amino acid residues are present in the SBP domains across the streptophytes, albeit with different conservations, indicating that those positions were fixed very early during land plants evolution.

The evolutionary analysis across streptophytes highlights the expansion of the SPL gene family in land plants, with different phylogenetic groups showcasing variations in the gene number and conserved motifs composition. The presence of unique motifs in specific phylogenetic groups, such as bryophyte-unique motifs in Group 1 proteins, suggests lineage-specific processes or functional roles. Group 2 proteins consists of a characteristic motif associated with miR156/529 regulation, but with variations in certain species that might impact miRNA targeting efficiency. Group 3 contains a high number of conserved motifs, including ankyrin motif, suggesting their involvement in protein–protein interactions. Additionally, we observed that the SPL proteins showed a similar pattern of conserved motifs between streptophyte algae, bryophytes and *A. thaliana* in Groups 2 and 4 (Fig. 3), with the exception of hornworts AaSPL3 and ApSPL3 proteins. However, in Group 1 the SPL proteins differed explicitly between analyzed plant species with SPL proteins from liverworts and mosses being more similar to each other than hornwort SPL proteins in the bryophyte lineage. Similar situation is observed between SPL Group 1 proteins within the tracheophytes lineage where all angiosperm proteins are shorter in comparison to the fern SPLs. Only the SBP domain was found to be a common motif for all SPL proteins regardless of the streptophyte lineage. Along with the SBP domain, we found additional motifs in the analyzed SPL proteins which especially in Group 3 and Group 4 showed high conservation between evolutionary distant plant species (Fig. 3). The function of these motifs is yet unknown, however, because of their high evolutionary conservation they might be considered as structural units important for proper function of encoded SPL proteins. The SBP domain is crucial for specific recognition and binding to *cis*-elements in the promoter of nuclear genes to regulate their expression. However, the additional conservation within the C-terminal part of those proteins may indicate that these conserved motifs are important for the Group 3 and Group 4 SPL proteins to orchestrate the proper expression profile in different tissues and organs throughout the plant life cycle. This could be achieved by interaction of these SPLs with other proteins via conserved C-terminal localized motifs, for example the ankyrin repeats which are known to be involved in protein–protein interactions. Still, the significance of these conserved motifs remains unknown and needs to be further investigated, especially using cross species studies.

Furthermore, gene structure analysis revealed that *SPL* genes across streptophyte algae and embryophytes display variations in exon–intron patterns. Notably, *SPL* genes from bryophytes and *Arabidopsis* share similar exon–intron organization within the same phylogenetic group with the exception of *A. agrestis SPL* genes from Group 3 and Group 4. Hornworts *SPL* genes from Group 3 and Group 4 possess only one or two very short introns in comparison to the complex structures of *SPL* genes from the liverwort *M. polymorpha,* moss *P. patens* and dicot *A. thaliana* (Fig. 4). To conclude, evidence based on available genomic data indicates the conservation of exon–intron structures within *SPL* clades with only slight variation in the number of exons and introns mostly observed in hornworts. This conservation is observed even between distantly related species like liverwort *M. polymorpha* and angiosperm *A. thaliana.* However, exceptions to this rule of *SPL* gene structure conservation can be found, like in *A. agrestis,* which can be related to the genome composition and structure.

The promoter region composition is a key element involved in the regulatory control of gene expression in a tissue specific manner or in response to different stimuli. Many *cis*-elements were found in the promoter regions of *SPL* genes from analyzed bryophytes and *A. thaliana,* mostly associated with growth and development, light, hormone, and stress responsiveness (Fig. 5). This data indicates that in each of the studied plant species, the *SPL* family is under complex and elaborate control of the transcription, regulated by various environmental and developmental changes. Interestingly, no similar set of *cis*-elements distribution was observed in the promoter regions of *SPLs* within the same phylogenetic group implying that the alteration of *cis*-regulatory elements took place during the land plants *SPL* genes evolution.

In order to further explore the expression landscape of *SPL* genes from the selected plant species, the expression profiles of investigated *SPL* genes were analyzed from different developmental stages and organs of each plant (Fig. 6). The obtained heat maps of expression profiles revealed that both bryophytes and *A. thaliana SPL* genes from phylogenetic Group 3 and Group 4 exhibit constitutive expression while *SPLs* belonging to Group 1 and Group 2 are expressed in a developmentally specific way or their expression is higher in specific organs/tissues. This differentiated expression pattern correlates with the posttranscriptional expression regulation by miR156 or miR529 family members of all genes from Group 2 (Fig. 1). miR156 is conserved across all land plant lineages while miR529 is mostly present in bryophytes and monocots. Although we did not find any proof of miR156 and miR529 presence in the genomes of investigated *A. agrestis* and *A. punctatus* species, our analysis revealed that the conserved miR156/529-responsive element in *AaSPL2* and *ApSPL2* genes can be recognized. Thus, it is highly likely that at least one of these miRNAs is present in the investigated hornwort species, especially since in another species, *A. angustus*, miR156 has been identified^28^.

Interestingly, *M. polmorpha* Mp*SPL1* is also regulated by miRNA, however by liverwort specific Mpo-MR-13^64,65^. Based on transcriptomic studies it was suggested that this Mpo-MR-13–Mp*SPL1* module might be involved in controlling the transition from vegetative to reproductive life cycle. Characteristic expression pattern of Mp*SPL1* has been observed with an explicit expression peak in gametangiophores along with simultaneous down-regulation of Mpo-MR-13 precursors at this developmental stage^80^. However, recent functional studies revealed a role of this Mpo-MR-13–Mp*SPL1* module in the regulation of meristem dormancy with superior control of this module by PIF-mediated phytochrome signaling^65^. Therefore, it cannot be excluded that the Mpo-MR-13–Mp*SPL1* module may play a dual role during *M. polmorpha* life cycle. Our analysis together with the literature data indicate that the miRNA–*SPL* regulatory module appeared very early during land plant evolution. It seems that this miRNA-mediated expression regulation for *SPL* genes from Group 2 is conserved in land plants while for liverwort Group 1 it may resemble lineage-specific mechanism.

## Conclusions

In summary, this study reports for the first time phylogenetic and diversification studies of the *SPL* gene family members from representatives of major streptophytes lineages. Streptophyte algae, liverworts and hornworts encode a minimal set of SPL proteins, which most probably resembles an archetype of *SPL* genes present in the ancestor of today's land plants from which all other *SPL* members might have originated. From our analysis we proposed four phylogenetic *SPL* groups with Group 3 and 4 being sister to Group 1 and 2. Only the SBP domain is a common feature identified for all SPL proteins regardless of the streptophyte lineage. However, depending on the phylogenetic group, SPL proteins may exhibit a group-specific or lineage-specific pattern of conserved motifs. Using three bryophytes and one angiosperm transcriptomic data, two distinct expression patterns were revealed for the *SPL* family members. We observed that mostly the miRNA-targeted *SPL* genes were expressed in a developmentally specific manner while the non-targeted *SPL* genes exhibited constitutive expression, suggesting their primary role in maintaining basal cellular functions. Our study emphasizes the importance of research on the biological relevance of *SPL* genes from different lineages of streptophytes representatives to provide a better understanding of the *SPL* family evolution and function.

### Supplementary Information

Supplementary Figures.

Supplementary Tables.

## Data Availability

Data associated with the manuscript are openly available at Zenodo: 10.5281/zenodo.7708436.
