# Supplementary material for: Comparative analysis of SPL transcription factors from streptophyte algae and embryophytes reveals evolutionary trajectories of SPL family in streptophytes
Source: Sci Rep. 2024 Jan 18;14:1611. doi: 10.1038/s41598-024-51626-2 (PMC10796333; doi:10.1038/s41598-024-51626-2)
Supplement: Supplementary file 1 — Supplementary Figures. [file 41598_2024_51626_MOESM1_ESM.docx]

**Comparative analysis of SPL transcription factors from streptophyte algae and embryophytes reveals evolutionary trajectories of *SPL* family in streptophytes**

**Alisha Alisha, Zofia Szweykowska-Kulinska, Izabela Sierocka***

Department of Gene Expression, Institute of Molecular Biology and Biotechnology, Faculty of Biology, Adam Mickiewicz University, Poznan, Poland, Uniwersytetu Poznanskiego 6, 61-614 Poznan, Poland

***Correspondence:** Izabela Sierocka, [izapaste@amu.edu.pl](mailto:izapaste@amu.edu.pl)

**Supplementary Figures**


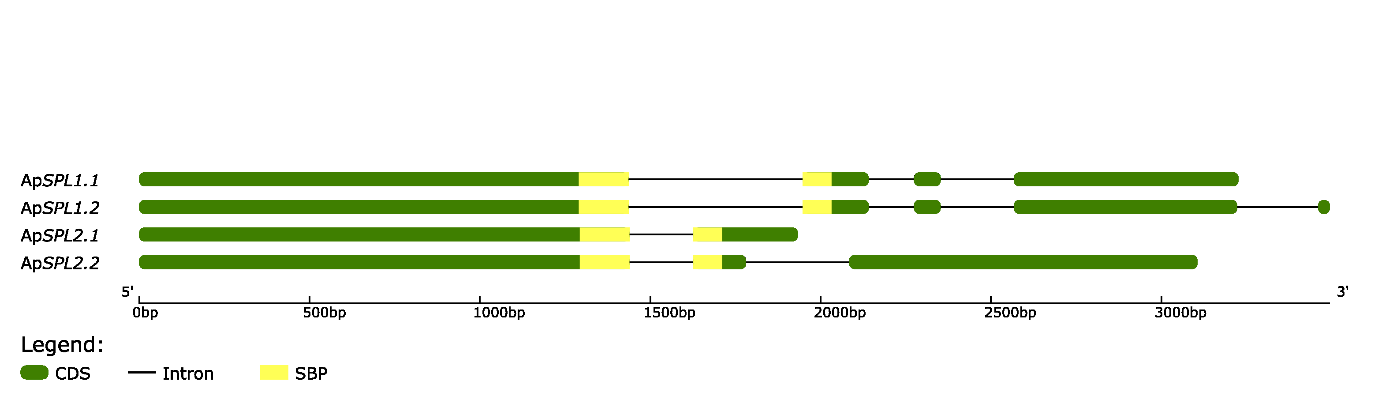


**Supplementary Figure 1** Diagram of exon-intron organization of Ap*SPL1* and Ap*SPL2* gene transcript isoforms from hornwort *Anthoceros punctatus*. The gene structures were analyzed using gene structure display server 2.0 [^53^](https://paperpile.com/c/fJQbk6/eRONk) and grouped based on their phylogenetic relationships. In each gene model, exons are shown as green boxes, introns as black lines and SBP-box as yellow rectangular shading. The scale shown at the bottom represents gene lengths in base pairs.


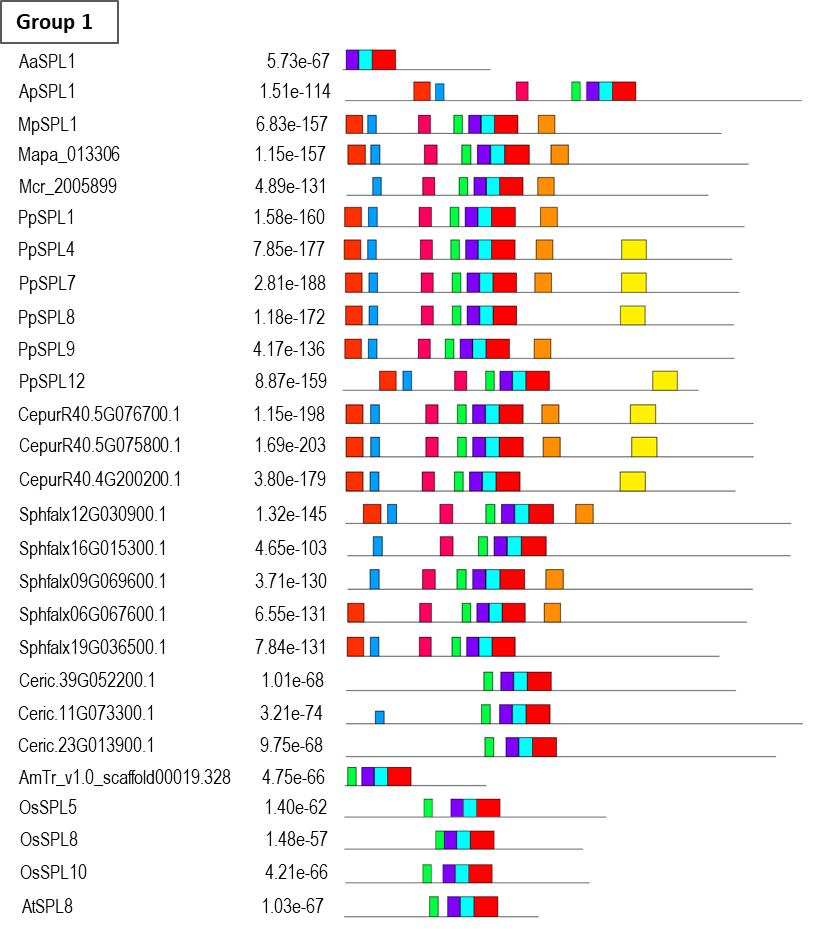


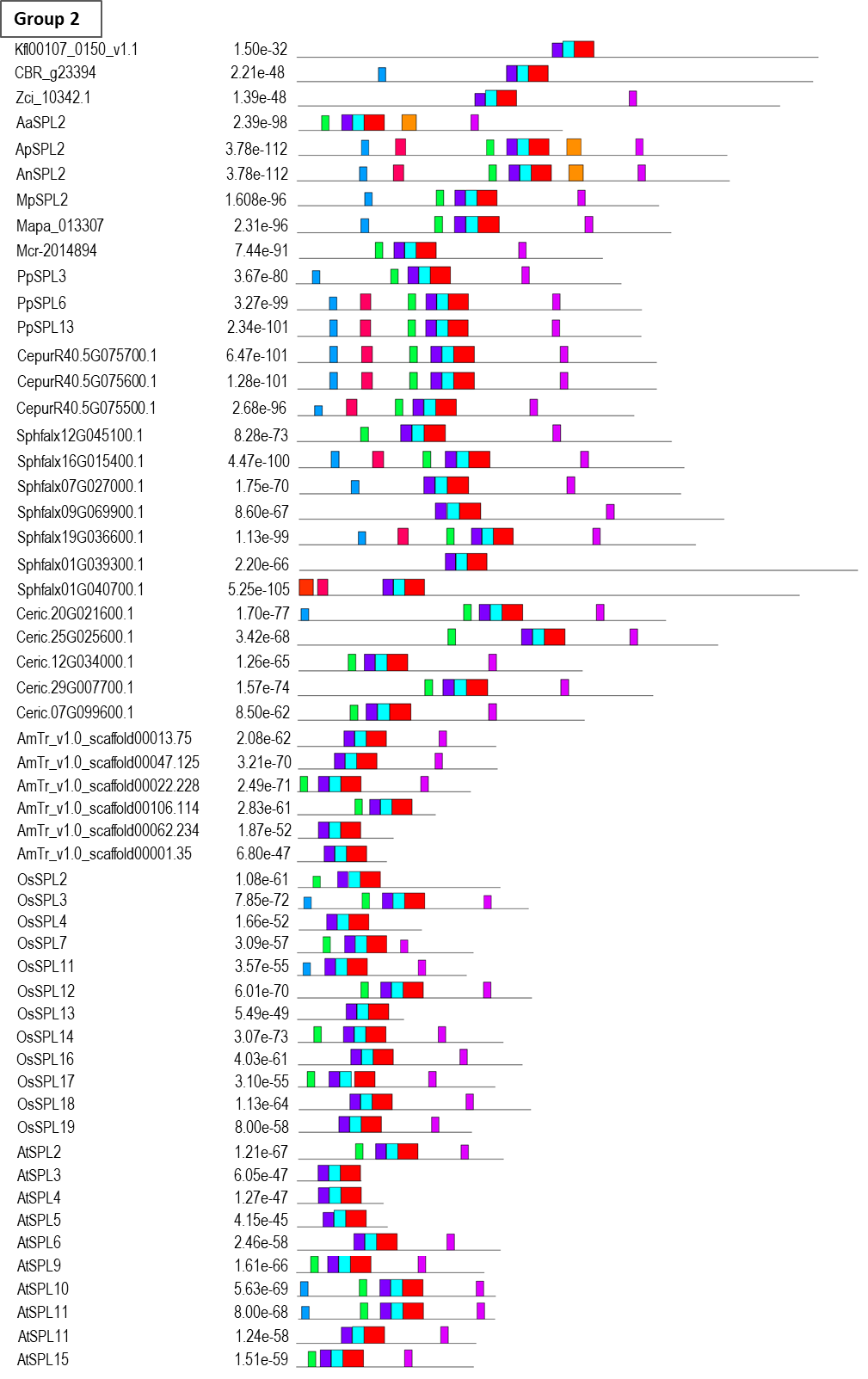


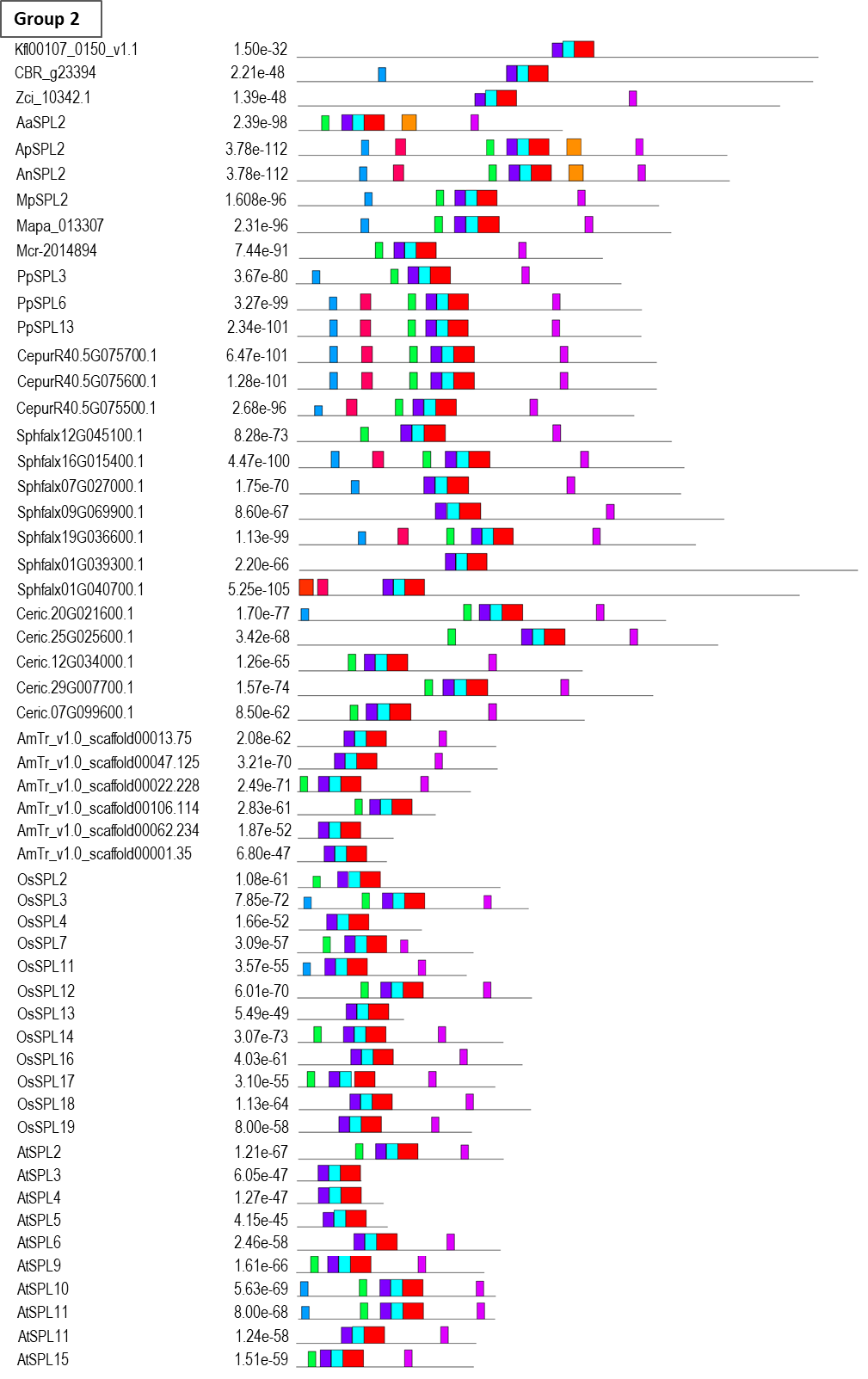


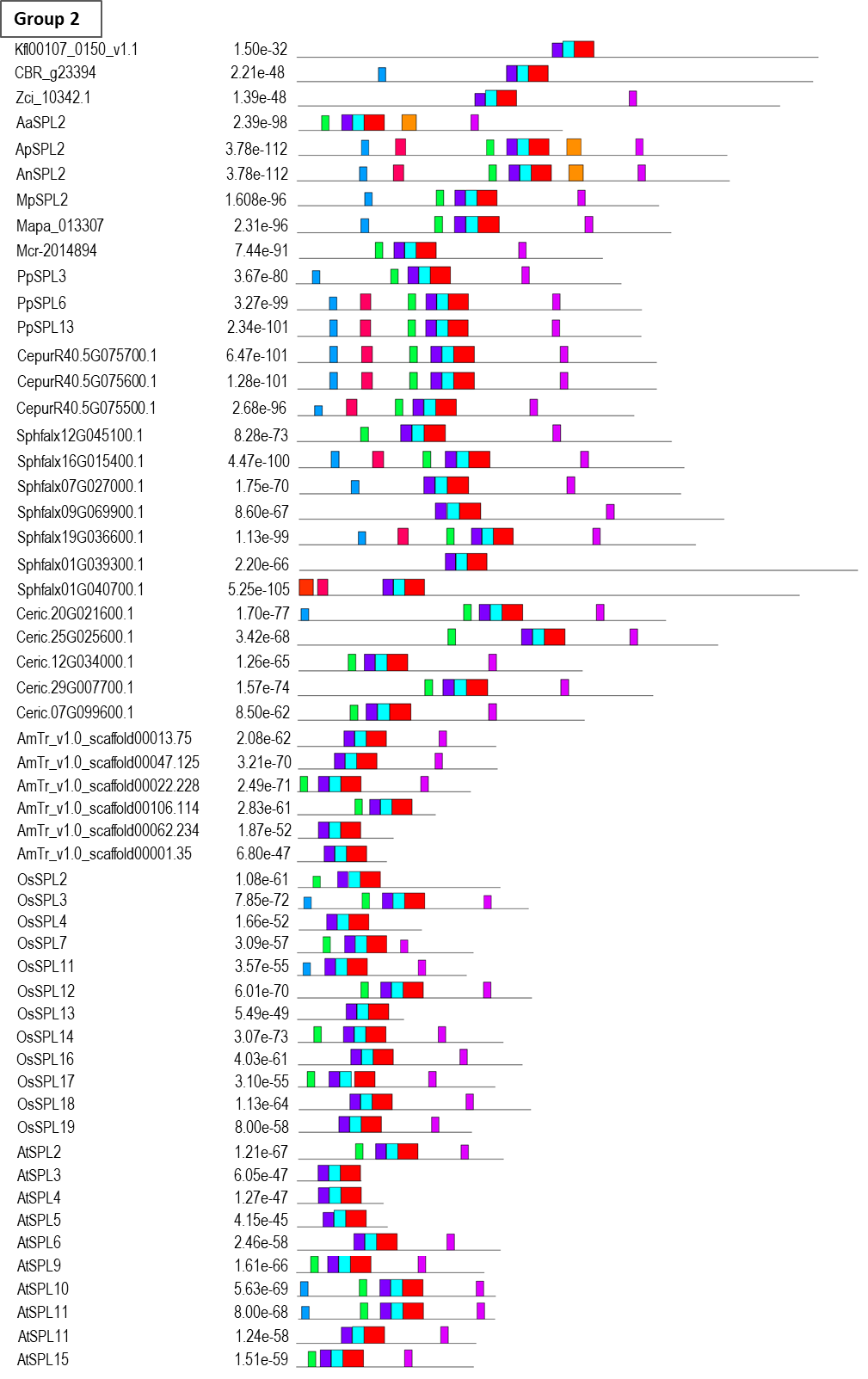


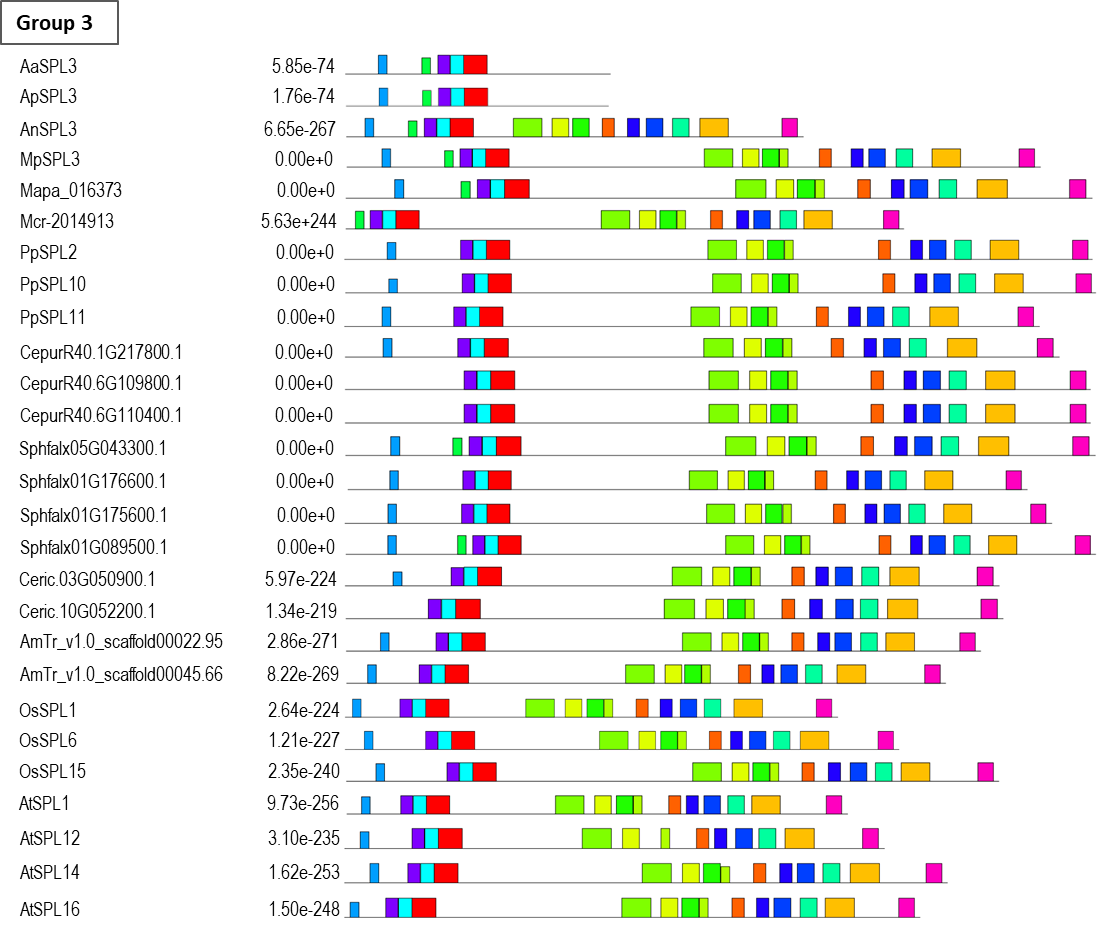


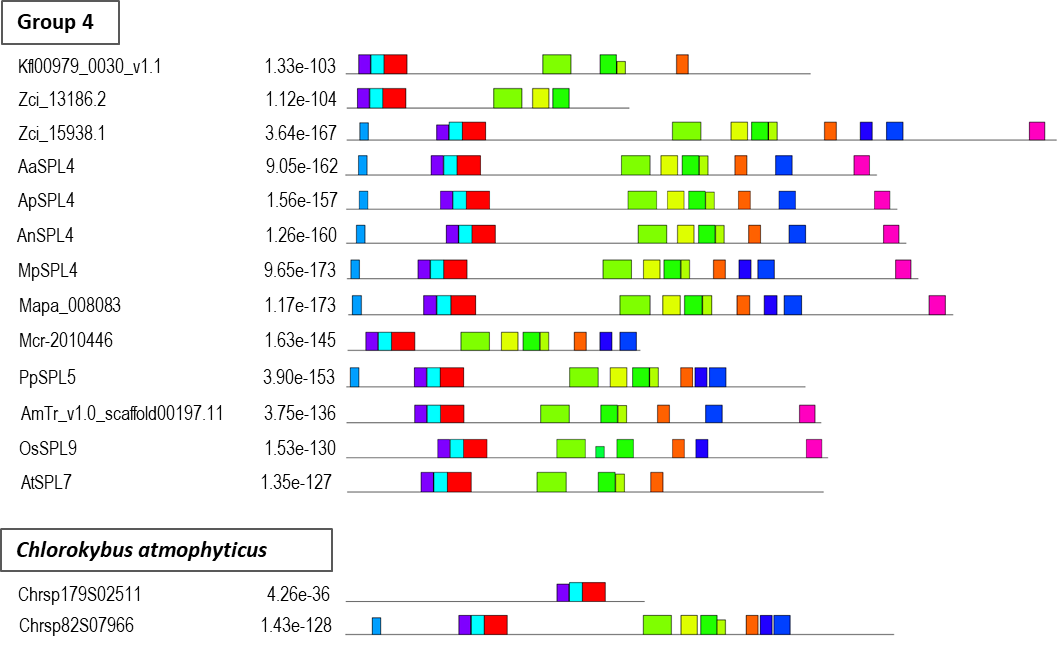


**Supplementary Figure 2** Conserved motifs in SPL proteins from representatives of streptophytes algae (*Chlorokybus atmophyticus, Klebsormidium nitens, Chara braunii* and, *Zygnema circumcarinatum*), liverworts (*Marchantia polymorpha, Marchantia paleacea* and, *Metzgeria crassipilis*), mosses (*Physcomitrium patens, Ceratodon purpureus* and, *Sphagnum fallax*), hornworts (*Anthoceros angustus, Anthoceros agrestis* and, *Anthoceros punctatus*), fern (*Ceratopteris richardii*) and, angiosperms (*Amborella trichopoda, Arabidopsis thaliana* and, *Oryza sativa*)*.* The SPL proteins are clustered into individual groups according to phylogenetic tree in Figure 1. The motif search was performed using MEME online tool [^54^](https://paperpile.com/c/fJQbk6/lG5fK) with full length protein sequences as a query. SPL proteins are grouped according to their phylogenetic relationships. Different motifs are represented with colors shown in the legend. Motifs 1, 2 and 4 with red, blue and violet color denote SBP-box domain which is conserved amongst all SPL proteins. The consensus sequence of each motif is presented in Table S3.


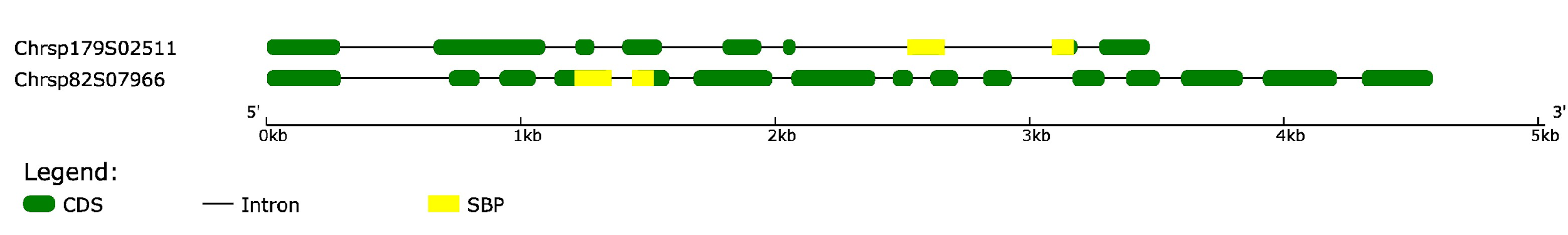


**Supplementary Figure 3:** Diagram of exon-intron organization of the *SPL* gene family from streptophytes alga *Chlorokybus atmophyticus*. The gene structures were analyzed using gene structure display server 2.0 [^53^](https://paperpile.com/c/fJQbk6/eRONk) and grouped based on their phylogenetic relationships. In each gene model, exons are shown as green boxes, introns as black lines and SBP-box as yellow rectangular shading. The scale shown at the bottom represents gene lengths in base pairs.
